# Novel *MSX1* variants identified in families with nonsyndromic oligodontia

**DOI:** 10.1038/s41368-020-00106-0

**Published:** 2021-01-08

**Authors:** Jinglei Zheng, Miao Yu, Haochen Liu, Tao Cai, Hailan Feng, Yang Liu, Dong Han

**Affiliations:** 1 Department of Prosthodontics, Peking University School and Hospital of Stomatology & National Engineering Laboratory for Digital and Material Technology of Stomatology & Beijing Key Laboratory of Digital Stomatology, Beijing, China; 2 National Institute of Dental and Craniofacial Research, NIH, Bethesda, MD USA

**Keywords:** Genetics, Diseases

## Abstract

The goal of this study was to identify *MSX1* gene variants in multiple Chinese families with nonsyndromic oligodontia and analyse the functional influence of these variants. Whole-exome sequencing (WES) and Sanger sequencing were performed to identify the causal gene variants in five families with nonsyndromic oligodontia, and a series of bioinformatics databases were used for variant confirmation and functional prediction. Phenotypic characterization of the members of these families was described, and an in vitro analysis was performed for functional evaluation. Five novel *MSX1* heterozygous variants were identified: three missense variants [c.662A>C (p.Q221P), c.670C>T (p.R224C), and c.809C>T (p.S270L)], one nonsense variant [c.364G>T (p.G122*)], and one frameshift variant [c.277delG (p.A93Rfs*67)]. Preliminary in vitro studies demonstrated that the subcellular localization of MSX1 was abnormal with the p.Q221P, p.R224C, p.G122*, and p.A93Rfs*67 variants compared to the wild type. Three variants (p.Q221P, p.G122*, and p.A93Rfs*67) were classified as pathogenic or likely pathogenic, while p.S270L and p.R224C were of uncertain significance in the current data. Moreover, we summarized and analysed the *MSX1*-related tooth agenesis positions and found that the type and variant locus were not related to the severity of tooth loss. Our results expand the variant spectrum of nonsyndromic oligodontia and provide valuable information for genetic counselling.

## Introduction

Congenital tooth agenesis (TA) is one of the most common dental anomalies and may lead to masticatory dysfunction, speech alteration, aesthetic problems, and malocclusion.^1^ Based on the existence of other ectodermal symptoms, TA can be classified as syndromic or nonsyndromic. Nonsyndromic TA can be further categorized into hypodontia (absence of one to five permanent teeth, excluding the third molars), oligodontia (absence of six or more permanent teeth, excluding the third molars), and anodontia (complete absence of teeth), based on the number of missing teeth.^2^ The overall incidence of TA is reported to be approximately 2–10%, excluding the third molars,^3,4^ whereas oligodontia is observed in approximately 0.08–0.16% of the population.^5^ TA has been associated with many factors, including genetics, epigenetics, and environmental factors. Among them, genetic defects have been demonstrated to play a major role.^6^

A number of genes have been shown to be associated with nonsyndromic TA, including axin inhibition protein 2 (*AXIN2*), ectodysplasin A (*EDA*), low-density lipoprotein receptor-related protein 6 (*LRP6*), muscle segment homeobox 1 (*MSX1*), paired box 9 (*PAX9*), and wingless-type mouse mammary tumour virus integration site family member 10A (*WNT10A*) and 10B (*WNT10B*). Variants in the above seven genes account for approximately 90% of the variants responsible for nonsyndromic TA.^7^ As the first gene identified in a family with TA,^8^ the *MSX1* gene is a hotspot candidate.

The *MSX1* gene is located on 4p16.2, belongs to the homeobox gene family, and encodes a transcription factor, MSX1.^9^ MSX1 has a highly conserved and important domain called the homeodomain (HD), which is encoded by exon 2 of the gene.^10^ *MSX1* plays crucial roles in regulating epithelial–mesenchymal interactions that are required for the morphogenesis of a variety of embryonic tissues, such as the limb bud, embryonic tail, hair follicle, and tooth bud.^11^ This molecule also has roles in regulating the development of the nervous system and tumour growth inhibition.^12^ *Msx1*^*−/−*^ mice are characterized by a deficiency of both mandibular and maxillary alveolar bones, a complete cleft palate, and a failure of tooth development, which is arrested at the bud stage.^13,14^ In humans, *MSX1* is associated with nonsyndromic TA (OMIM, #106600—Tooth Agenesis, Selective, 1; STHAG1), nonsyndromic cleft lip with or without cleft palate (CL/P, OMIM #608874, Orofacial Cleft 5, OFC5), Witkop syndrome (OMIM, #189500), and Wolf–Hirschhorn syndrome (WHS, OMIM, #194190).^12^ To date, according to the Human Gene Mutation Database (HGMD, http://www.hgmd.cf.ac.uk/) and PubMed database, 36 variants have been identified in the *MSX1* gene that contribute to nonsyndromic TA, and most of them are missense or frameshift variants.^1,6,8,10,15–39^

This study aimed to continue to expand the variant spectrum of *MSX1* associated with nonsyndromic oligodontia, explore the functional impacts of the identified novel variant loci, and analyse the genotype–phenotype correlations.

## Results

### Clinical findings and variant detection

**Fig. 1:**
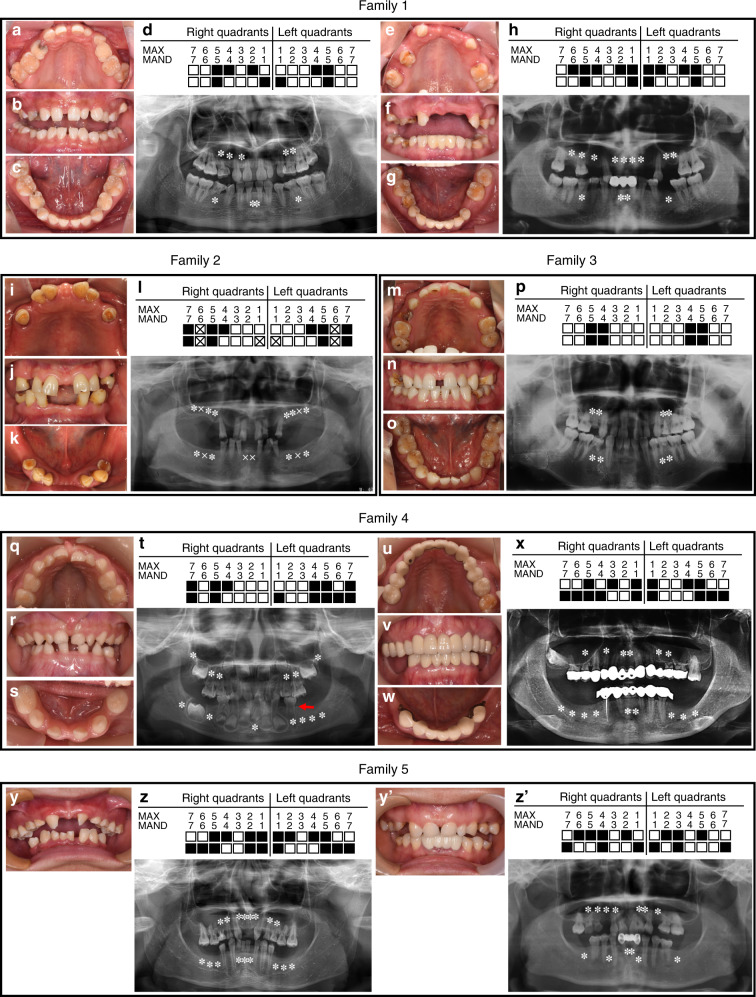
Dental characteristics of five families with nonsyndromic oligodontia with MSX1 variants. **a**–**h** Digital photographs, panoramic radiographs, and schematics of oligodontia in family 1, including proband #1 and her mother. **i**–**l** Digital photographs, a panoramic radiograph, and a schematic of oligodontia in family 2, including proband #2. **m**–**p** Digital photographs, a panoramic radiograph, and a schematic of oligodontia in family 3, including proband #3. **q**–**x** Digital photographs, panoramic radiographs, and schematics of oligodontia in family 4, including proband #4 and his mother. The red arrow indicates taurodontism of the left mandibular first primary molar. **y**–**z’** Digital photographs, panoramic radiographs, and schematics of oligodontia in family 5, including proband #5 and his mother. Asterisks and solid squares indicate congenitally missing teeth. Crosses and squares with crosses indicate the extracted teeth

**Fig. 2:**
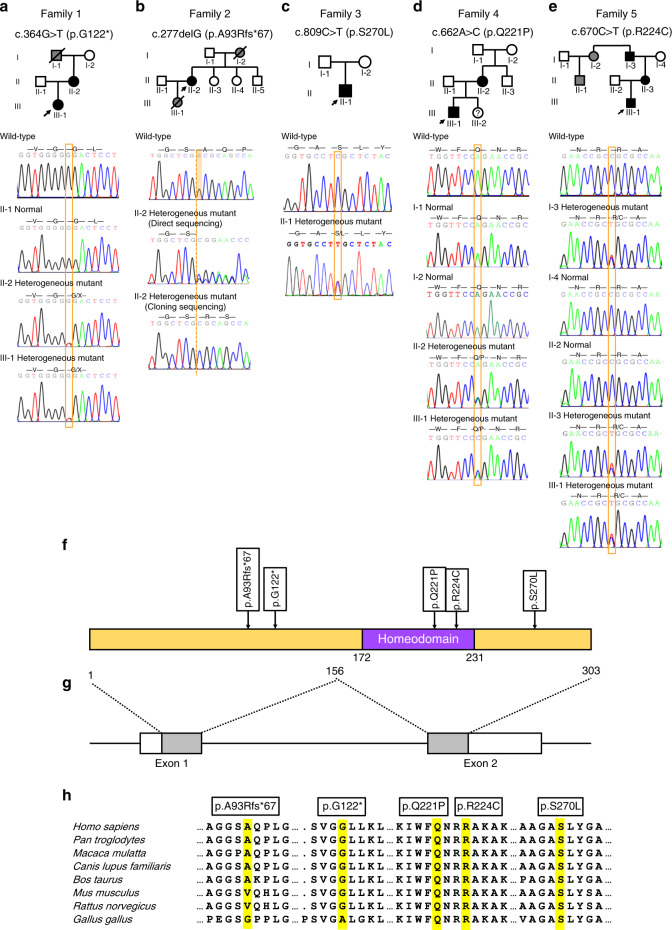
Sequencing chromatograms of five families and conservation analysis of the MSX1 variants. **a** Pedigree of Family 1 with nonsyndromic oligodontia. Available DNA sequencing chromatogram showing a heterozygous *MSX1* variant of c.364G>T (p.G122*) in proband #1 (III-1) and her mother (II-2). **b** Pedigree of Family 2 with nonsyndromic oligodontia. Available DNA sequencing chromatogram showing a heterozygous *MSX1* variant of c.277delG (p.A93Rfs*67) in proband #2 (II-2). **c** Pedigree of Family 3 with nonsyndromic oligodontia. Available DNA sequencing chromatogram showing a heterozygous *MSX1* variant of c.809C>T (p.S270L) in proband #3 (II-1). **d** Pedigree of Family 4 with nonsyndromic oligodontia. Available DNA sequencing chromatogram showing a heterozygous *MSX1* variant of c.662A>C (p.Q221P) in proband #4 (III-1) and his mother (II-2). **e** Pedigree of Family 5 with nonsyndromic oligodontia. Available DNA sequencing chromatogram showing a heterozygous *MSX1* variant of c.670C>T (p.R224C) in proband #5 (III-1), his mother (II-3), and his grandfather (I-3). **f** Schematic diagram of the MSX1 protein structure showing the localization of five novel variants identified in these TA pedigrees. **g** Schematic diagram of the *MSX1* gene. **h** Alignment conservation analysis of the MSX1 amino acid sequences among different species. Black arrows indicate the proband in each family. Black squares and circles represent TA patients. Grey squares and circles represent individuals with TA, but their DNA samples were not available. Squares and circles with a slash represent individuals who have passed away. A circle with a question mark represents an individual who is too young to be confirmed

The TA patterns (excluding the third molars) and pedigrees of five Chinese families are shown in Figs. 1 and 2. Using whole-exome sequencing (WES), we identified five novel heterozygous variants of the *MSX1* gene in these families and confirmed them by Sanger sequencing (Fig. 2a–e). Variants in families 1–4 were not identified in the 1000 Genomes, Exome Aggregation Consortium (ExAC), or gnomAD databases (Table 1).

**Table 1:** Harmful prediction of the five novel variants in *MSX1*

Families	Exon	Nucleotide change	Protein change	Variation type	ExAC (MAF)	SIFT	PolyPhen-2	Mutation Taster	ACMG classification (evidence of pathogenicity)
Family 1	1	c.364G>T	p.G122*	Nonsense	—			Disease causing	Pathogenic (PVS1 + PM2 + PM5 + PP1)
Family 2	1	c.277delG	p.A93Rfs*67	Frameshift	—			Disease causing	Pathogenic (PVS1 + PM2 + PP3)
Family 3	2	c.809C>T	p.S270L	Missense	—	0.057 (tolerated)	0.213 (benign)	Disease causing	Uncertain significance (PM2 + PP3)
Family 4	2	c.662A>C	p.Q221P	Missense	—	0.032 (damaging)	1.000 (probably damaging)	Disease causing	Likely Pathogenic (PS2 + PM2 + PP1 + PP3)
Family 5	2	c.670C>T	p.R224C	Missense	4.012 × 10^−6^	0.000 (damaging)	0.998 (probably damaging)	Disease causing	Uncertain significance (PP1 + PP3)

Note: — variant was not found in ExAC

ACMG, American College of Medical Genetics; PM, pathogenic criterion is weighted as moderate; PP, pathogenic as strong; PVS, pathogenic criterion is weighted as very strong

In family 1, proband #1 was an 18-year-old girl in good health. Clinical and radiographic examinations revealed the absence of nine permanent teeth and the presence of few deciduous teeth. The maxillary central incisors were narrow, and the left maxillary lateral incisor was cone-shaped (Fig. 1a–d). Clinical and panoramic diagnosis revealed that her mother also suffered from oligodontia with the absence of 13 permanent teeth (Fig. 1e–h). The mother declared that the proband’s grandfather also suffered from TA, while the other family members had normal dentition. The entire family had normal hair, skin, and nails, without any other syndromic phenotypes. WES and Sanger sequencing revealed that proband #1 (III-1) and her mother (II-2) carried the same *MSX1* heterozygous nonsense variant, c.364G>T (p.G122*) (Fig. 2a). According to family cosegregation, the variant of the proband was inherited from his mother through an autosomal-dominant mode of inheritance.

In family 2, proband #2 was a 55-year-old woman in good health. According to the dental medical records, all the first molars and mandibular central incisors were lost due to extraction. Clinical and radiographic examinations revealed that she had ten congenitally missing permanent teeth (Fig. 1i–l). She declared that her mother and daughter also suffered from TA, while other family members had normal dentition. WES and Sanger sequencing revealed that proband #2 (II-2) carried an *MSX1* heterozygous frameshift variant, c.277delG (p.A93Rfs*67) (Fig. 2b). Unfortunately, her mother and daughter had passed away and were unavailable for genetic screening.

In family 3, proband #3 was a 28-year-old man in good health. He denied any history of tooth extraction. Clinical and radiographic examinations revealed the absence of eight permanent teeth (Fig. 1m–p). His hair, skin, and nails appeared normal. According to the proband’s description, his parents had normal dentition. Unfortunately, they were unavailable for genetic screening. WES and Sanger sequencing revealed that proband #3 (II-1) carried an *MSX1* heterozygous missense variant, c.809C>T (p.S270L) (Fig. 2c). Based on the current sequencing results, the inheritance pattern and the pathogenicity of this variant could not be clearly defined.

In family 4, proband #4 was a 4-year-old boy in good health. Clinical and radiographic examinations revealed the absence of 13 permanent teeth (Fig. 1q–t) and the presence of taurodontism in his left mandibular first primary molar (Fig. 1t, red arrow). His mother also suffered from oligodontia, which was restored with maxillary and mandibular fixed bridges (Fig. 1u–w). A panoramic radiograph revealed that she lacked 15 permanent teeth (Fig. 1x). The proband and his mother showed normal development of hair, skin, nails, and facial appearance and denied any history of heat intolerance. The proband had a 1-year-old sister, but dental information and a DNA sample were not available because of her age (III-2). The mother stated that all other family members had normal dentition. WES and Sanger sequencing revealed that proband #4 (III-1) and his mother (II-2) carried an *MSX1* heterozygous missense variant, c.662A>C (p.Q221P), with an autosomal-dominant pattern of inheritance, while the grandparents of the proband (I-1 and I-2) had wild-type sequences (Fig. 2d). Family cosegregation demonstrated that the variant in the proband was inherited from his mother. Furthermore, we confirmed the genetic relationship between the proband’s mother (II-2) and grandparents (I-1 and I-2). Therefore, p.Q221P could be identified as a de novo variant.

In family 5, proband #5 was a 12-year-old boy with good physical health. His facial features, hair, skin, and nails appeared normal. Clinical and radiographic examinations revealed the absence of 17 permanent teeth (Fig. 1y–z). All remaining permanent teeth were smaller than normal, especially the bilateral maxillary first molars, which had a narrow occlusal surface in buccolingual directions and were similar to the deciduous teeth. His mother was missing 13 permanent teeth, which were restored with a fixed bridge on the lower anterior teeth (Fig. 1y’–z’). His mother stated that her father, aunt, and cousin had congenital missing teeth as well and denied any consanguineous marriages in the family. WES and Sanger sequencing revealed that proband #5 (III-1) carried an *MSX1* heterozygous missense variant, c.670C>T (p.R224C, rs1342784720). We sequenced all the available members in this family (I-3, I-4, II-2, and II-3) and found that the variant was carried by all the affected members (I-3 and II-3) but not by the unaffected members (I-4 and II-2) (Fig. 2e). Thus family cosegregation also revealed an autosomal-dominant inheritance pattern. This variant was found in the ExAC Database (minor allele frequency (MAF): 0.000004012) (Table 1) and Single Nucleotide Polymorphism Database (dbSNP) but had not been reported to be associated with any anomalies.

### Conservation and bioinformatics analysis

We conducted conservation and bioinformatics analyses to predict the effects of the five identified novel *MSX1* variants. We found that variants c.277delG (A93Rfs*67) and c.364G>T (G122*) were located in exon 1 of *MSX1*, and three missense variants, c.662A>C (Q221P), c.670C>T (R224C), and c.809C>T (S270L), were located in exon 2 of *MSX1* (Fig. 2f–g). Among them, Q221P and R224C were located in the highly conserved HD of MSX1, which was encoded by exon 2 (Fig. 2f–g). According to Sorting Intolerant from Tolerant (SIFT), polymorphism phenotyping (PolyPhen-2), MutationTaster, and the classification of pathogenic variants with the standards of the 2015 American College of Medical Genetics and Genomics and the Association for Molecular Pathology, p.G122* and p.A93Rfs*67 were predicted to be pathogenic, while p.Q221P was predicted to be likely pathogenic. However, the pathogenicity of p.S270L and p.R224C was of uncertain significance in the current data and requires further investigation (Table 1). The results of amino acid sequence alignment of the MSX1 protein among multiple species showed that Q221, R224, and S270 were highly evolutionarily conserved but A93 and G122 were not (Fig. 2h).

### Preliminary functional analysis

**Fig. 3:**
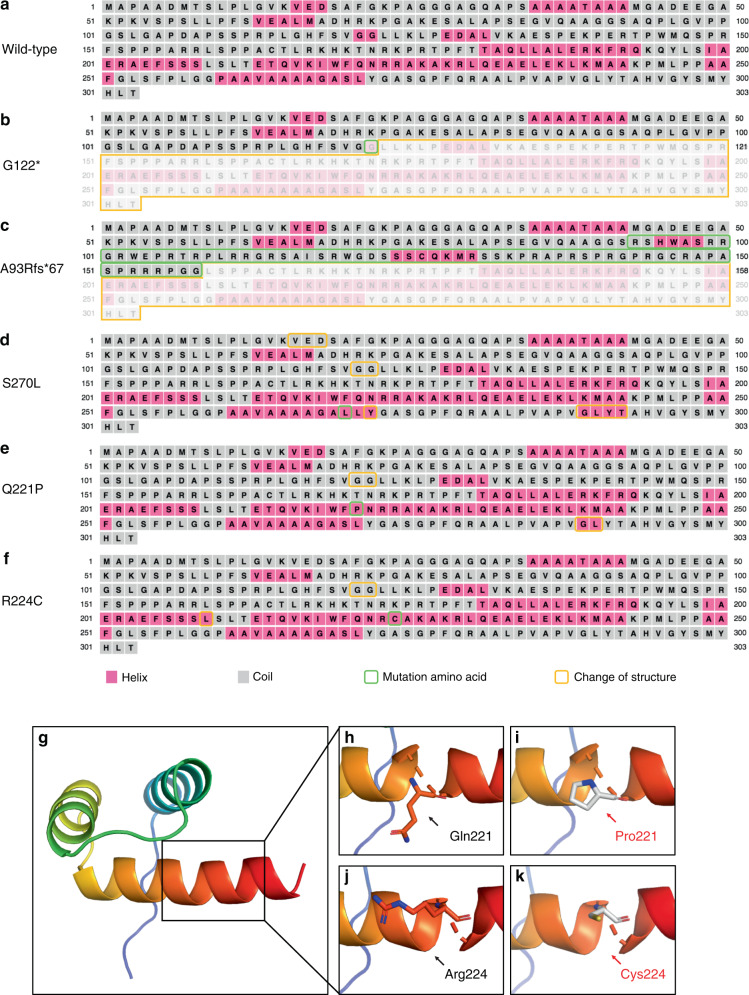
Secondary and tertiary structure analysis of mutated MSX1 protein. **a** The predicted secondary structure of the wild-type MSX1 protein. **b**–**f** The predicted secondary structure of five mutated MSX1 proteins. Sites of variants are indicated by green squares. The structural changes in these mutated proteins compared to the wild-type MSX1 are indicated by orange squares. α-Helices are represented as pink squares, while coils are represented as grey squares. **g** The predicted three-dimensional models of HD in MSX1. **h**–**k** The three-dimensional structural changes of two missense variants. The wild-type (**h**, **j**) and mutated residues (**i**, **k**) are shown in black and red, respectively

First, the secondary and tertiary protein structures were predicted to analyse the functional effects of *MSX1* variants (Fig. 3a–k). The nonsense variant G122* and frameshift variant A93Rfs led to truncated proteins, resulting in severe changes in the MSX1 secondary structure (Fig. 3b–c). Missense variants S270L, Q221P, and R224C led to the substitution of one amino acid and caused slight changes in the secondary structure (Fig. 3d–f). Tertiary structure analysis of the Q221P and R224C variants in the HD (Fig. 3g) showed that Q221P resulted in the substitution of a polar residue Gln221 with a nonpolar residue Pro (Fig. 3h–i), and R224C resulted in the substitution of a basic amino acid residue Arg224 with a polar residue Cys, which was much shorter than Arg (Fig. 3j–k).

**Fig. 4:**
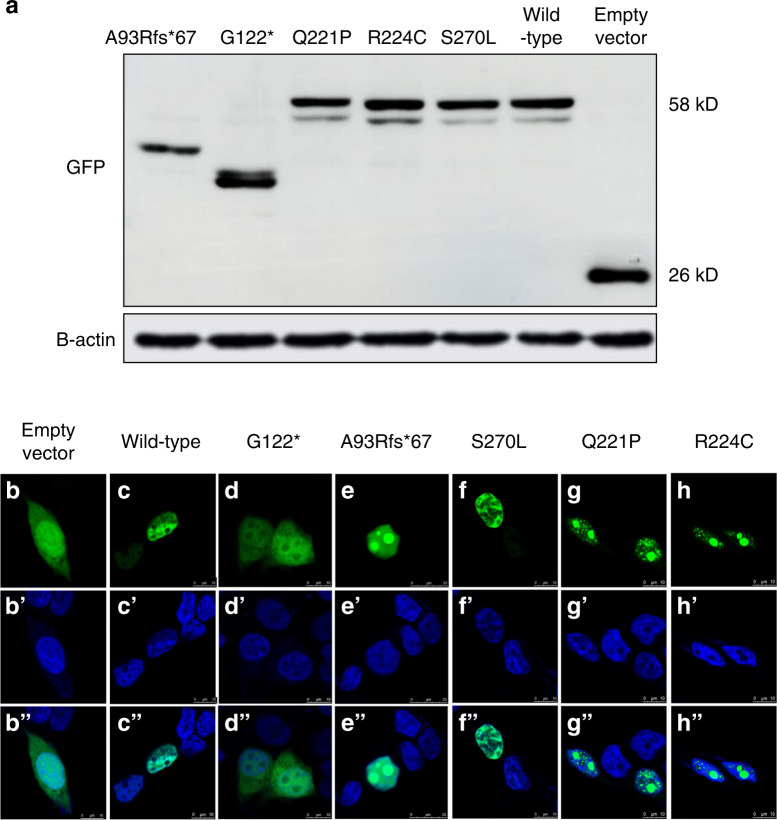
The expression and subcellular localization of wild-type and mutated MSX1 proteins. **a** Western blot analysis of total protein from wild-type or mutated MSX1 cells transfected into 293T cells using anti-GFP and anti-β-actin antibodies. Mutated proteins with normal length and truncated proteins were detected. An empty vector was transfected into cells as a negative control. **b**–**h** Subcellular localization of wild-type or mutated MSX1 in vitro. Cells transfected with GFP-G122* show expression in the entire cytoplasm with weak expression in the nucleus. GFP-A93Rfs*67 is detected in the nucleus with dense clumping. GFP-S270L is located in the same location as the wild type. GFP-Q221P and GFP-R224C show densely clumped distributions within the nucleus. The empty vector with GFP was transfected into 293T cells as a negative control. **b**’–**h**’ Nuclei stanning by DAPI, 4,6-diamino-2-phenylindole. **b**”**-h**” Merge of GFP and DAPI. MSX1 (GFP, green); nuclei (DAPI, blue)

Second, we performed in vitro functional analysis to evaluate the influence of the five identified novel variants. To test whether these five mutated plasmids could be expressed in vitro, we detected the wild-type and five mutated MSX1 proteins with green fluorescent protein (GFP) using an anti-GFP antibody. Western blotting and quantitative reverse transcription polymerase chain reaction (qRT-PCR) analysis showed that the wild-type and all five mutated proteins were expressed (Fig. 4a and Supplementary Fig. 1). A93Rfs*67 and G122* expressed truncated proteins, as predicted, while Q221P, R224C, and S270L expressed proteins of the same size as the wild type (Fig. 4a).

To evaluate the nuclear localization of the MSX1 variants, we determined the subcellular localization of the five mutated proteins by immunofluorescence. The results showed that wild-type MSX1 was located in the nucleus as a transcription factor (Fig. 4c–c”), but the localization of the MSX1 variants G122*, A93Rfs*67, Q221P, and R224C was altered. GFP-G122* was located in the whole cytoplasm with weak expression in the nucleus (Fig. 4d–d”). GFP-A93Rfs*67 showed a diffuse distribution in the nucleus and dense expression inside the nucleolus (Fig. 4e–e”). GFP-Q221P and GFP-R224C were scattered in the nucleus and densely clumped in the nucleolus (Fig. 4g–g”, h–h”). The results indicated that G122*, A93Rfs*67, Q221P, and R224C severely affected the nuclear localization of MSX1, which might contribute to the pathogenic process of oligodontia in our patients. However, the S270L variant seemed to be located in the same place in the cell as the wild type (Fig. 4f–f”), suggesting that the S270L variant might not affect the nuclear localization of MSX1.

### Statistical analysis of the *MSX1*-related TA pattern

**Fig. 5:**
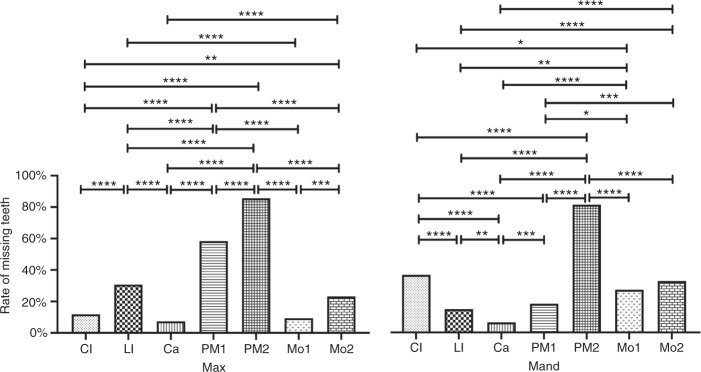
The pattern of MSX1-related tooth agenesis. The rate of missing teeth in 101 patients with *MSX1* variants was compiled at seven tooth positions in the maxillary and mandibular regions. A significant difference is marked by an asterisk (*P = 0.033 2, **P = 0.002 1, ***P = 0.000 2, and ****P < 0.000 1). CI, central incisor; LI, lateral incisor; Ca, canine; PM1, first premolar; PM2, second premolar; Mo1, first molar; Mo2, second molar

To analyse the specific TA pattern of nonsyndromic TA patients with *MSX1* variants, we compiled the number of congenital missing teeth of 101 patients (Supplementary Table 1) at each of the 7 positions in the 4 quadrants, excluding the third molars and tooth extractions (Table 2). We performed chi-square tests (or Fisher’s exact test) to determine the statistically significant differences between the different tooth positions (Fig. 5).

**Table 2:** The number of missing teeth in 101 patients with *MSX1* mutations

Quadrant	Tooth position							Total missing number
	1	2	3	4	5	6	7	
Max R	13/101	33/101	8/101	61/101	88/101	10/101	24/101	237/707
Max L	11/101	29/101	7/101	57/101	85/101	9/101	23/101	221/707
Mand R	36/101	17/101	5/101	17/101	80/101	26/101	34/101	215/707
Mand L	38/101	13/101	8/101	20/101	84/101	29/101	32/101	224/707
Max	24/202 (11.88%)	62/202 (30.69%)	15/202 (7.43%)	118/202 (58.42%)	173/202 (85.64%)	19/202 (9.41%)	47/202 (23.27%)	458/1414 (32.39%)
Mand	74/202 (36.63%)	30/202 (14.85%)	13/202 (6.44%)	37/202 (18.32%)	164/202 (81.19%)	55/202 (27.23%)	66/202 (32.67%)	439/1414 (31.05%)

Note: (1) The numerators indicate the number of missing teeth and the denominators indicate the number of individuals. (2) There was no statistically significant difference between the left and right arch, therefore the data for equivalent teeth on the left and right were combined at the bottom. (3) The number in the bracket indicates the rate of missing teeth

We found that the average number of *MSX1*-related missing teeth in 101 patients was 12.26 ± 3.06. The average incidence rate of missing teeth in the maxillary arch (32.39%) was comparable to that in the mandibular arch (31.05%) (*P* > 0.05). The rate of TA in the right quadrant (31.97%) was also similar to that in the left quadrant (31.47%) (*P* > 0.05). The maxillary second premolars (85.64%), mandibular second premolars (81.19%), and maxillary first premolars (58.42%) were the most frequently missing teeth. In contrast, the teeth with the lowest missing rates were mandibular canines (6.44%), maxillary canines (7.43%), and maxillary first molars (9.41%). Statistically significant differences in the missing tooth rate at different tooth positions are presented in Fig. 5 (*P* < 0.05).

Furthermore, statistical analysis of the relationship between *MSX1* variant domains and missing tooth positions was performed (Supplementary Figs. 2–4) based on the summary of all reported cases with nonsyndromic TA caused by the *MSX1* variant (Supplementary Table 1). Statistical results showed that missing tooth positions appeared to have no significant correlation with the variant domains (exon 1, HD in exon 2, and the rest of the region of exon 2).

## Discussion

Nonsyndromic TA caused by *MSX1* variants is inherited in an autosomal-dominant manner.^1^ To date, there are 36 *MSX1* variants associated with nonsyndromic TA, and most of them are missense (16/36) and frameshift variants (12/36). We found that, among these variants, 75% of the missense variants are located in the HD, while only 33% of the frameshift variants are located in the HD (Supplementary Table 1). In this study, we identified five novel *MSX1* variants in Chinese families with oligodontia inherited in an autosomal-dominant manner: three missense variants (Q221P, R224C, and S270L), one nonsense variant (G122*), and one frameshift variant (A93Rfs*67). All the missense variants were located in exon 2, while two of them were located in HD, thus supporting the hypothesis that missense variants are more likely to be found in areas of high evolutionary constraint^40^ and thereby suggesting that high conservation could be a marker for amino acids to be a hotspot for missense variants. However, for frameshift variants, the locations were not related to the conservation of amino acids.

Except for S270L, the other four variants showed different subcellular localizations of MSX1 compared to the wild type. As a highly conserved sequence, the HD is composed of an extended N-terminal arm and three α-helices, which contribute to structural stability, DNA binding specificity, transcriptional repression, and protein interactions.^41^ The HD contains two nuclear localization signal (NLS) sequences: NLS1 (_161_RKHKTNRKPR_170_) located upstream of the HD and NLS2 (_216_NRRAKAKR_223_) located downstream of the HD. NLS1 and NLS2 work cooperatively to mediate the nuclear localization of MSX1.^42^ Therefore, the presence or absence of HD variants may result in impaired or lost transcriptional nuclear localization of MSX1. Interestingly, only G112* was detected in the cytoplasm and nucleus, thereby suggesting that nuclear localization was impaired. Furthermore, the nucleolar expression of A93Rfs*67, Q221P, and R224C might be due to the opening of chromatin.^43^ In a previous study, a similar result was found for another MSX1 missense variant, R196P, which is located in HD.^44^ Notably, although S270L was predicted to cause a change in the MSX1 secondary structure, the subcellular localization of the S270L variant was not affected. A possible explanation is that S270L is located outside of the HD and does not change the HD structure or nuclear localization. Based on the current results, S270L was identified to have uncertain significance. Whether the variant is pathogenic requires further analysis. Moreover, the G122* variant was mainly located in the cytoplasm, thus indicating that this nonsense variant resulted in a truncated MSX1 protein without an HD and severely affected the nuclear translocation process of the MSX1 variant. Although the frameshift variant A93Rfs*67 altered the subsequent sequence after the 93rd amino acid, resulting in a truncated MSX1 without HD, the A93Rfs*67 variant was detected in the nucleus with dense clumping. This finding might be because the 67 mutated amino acids may have formed a structure similar to that governing MSX1 nuclear localization in HD. However, further structural and functional experiments are required to elucidate the exact mechanism.

Analysis of the missing tooth positions in 101 patients with *MSX1* variants revealed that the maxillary second premolar was the most vulnerable, followed by the mandibular second premolar and maxillary first premolar, thereby suggesting that *MSX1* is crucial for premolar development. Our findings were consistent with previous studies.^19,45^ Moreover, we found that the maxillary and mandibular canines and the maxillary first molars were least affected. This finding had not been previously reported, suggesting that *MSX1* might be nonessential during the development of these teeth.

In conclusion, the results from our study broaden the variant spectrum of *MSX1*. Information on *MSX1*-related TA patterns offers new evidence for genotypic studies and can guide health care providers in diagnosis, treatment, and genetic counselling.

## Methods

### Pedigrees and patients

All the probands and family members of five pedigrees requested treatment advice at the Department of Prosthodontics, Peking University School of Stomatology (Beijing, China) due to their congenital missing teeth. Oral examinations and panoramic dental radiographs were taken to reveal the exact number of missing teeth and other deficiencies. The medical history and physical examinations of the patients confirmed the absence of previous tooth extractions or other developmental abnormalities. Informed consent was obtained from all the participants. This study was approved by the Ethics Committee of Peking University School and Hospital of Stomatology (PKUSSIRB-201736082).

### WES, bioinformatics, and conservation analysis

Genomic DNA from these five probands was isolated from peripheral blood lymphocytes using a BioTek DNA Whole-blood Mini Kit (BioTek, Beijing, China) according to the manufacturer’s instructions. WES was conducted by Beijing Angen Gene Medicine Technology (Beijing, China) using DNA from the patients to identify potential pathogenic variants.

A series of bioinformatics databases, such as 1000 Genomes, ExAC, dbSNP, SIFT, PolyPhen-2, and MutationTaster, were used for variant screening and confirmation as well as to predict pathogenicity. Based on the WES results, we filtered all nonsynonymous single-nucleotide variants and insertions/deletions according to the MAF ≤0.01 in the databases mentioned above. Next, we selected all the variants in the genes associated with TA based on reports from previous studies. Then the variants that affect protein function were predicted based on the results obtained from SIFT, PolyPhen-2, and MutationTaster.^46^ The secondary structure of wild-type MSX1 and five mutated variants was predicted using PsiPred 4.0 (http://bioinf.cs.ucl.ac.uk/psipred). An optimum template of the HD (from amino acids 175 to 228) was selected for homology modelling analysis through SWISS-MODEL (https://swiss-model.expasy.org). Therefore, the three-dimensional structural changes of Q221P and R224C variants were captured using the PyMOL software (Molecular Graphics System, DeLano Scientific, CA).

For conservation analysis, alignment of the MSX1 amino acid sequence among multiple species was conducted using ClustalX 2.1. The MSX1 amino acid sequences of different vertebrate species were obtained from the National Center for Biotechnology Information.

### Sanger sequencing and clone sequencing

For verification of the WES results, the related *MSX1* (NM_002448) fragments were sequenced using Sanger sequencing. Genomic DNA from these family members was isolated according to the procedure described above. The primers for two exons of *MSX1*, *MSX1*-1F (5’-CTGGCCTCGCCTTATTAGC-3’) and *MSX1*-1R (5’-GCCTGGGTTCTGGCTACTC-3’) and *MSX1*-2F (5’-TGGCGGCACTCAATATCTGG-3’) and *MSX1*-2R (5’-CAGATCTGTCGTGGGTGTTCA-3’), were specifically designed to detect variants. The exonic region of the *MSX1* gene of five families was amplified using PCR with Taq PCR Master Mix (BioTek, Beijing, China). The PCR products were sequenced by Tsingke Biological Technology (Beijing, China). Once the frameshift variant was detected, the PCR product harbouring this variant was cloned into the pClone007 Simple Vector (Tsingke, Beijing, China) to identify the exact status of the variant.

### Construction of plasmids

The full-length coding region of the human *MSX1* gene was cloned into the pEGFP-C1 expression vector with enhanced GFP to synthesize the wild-type plasmid pEGFP-C1-MSX1. In vitro site-directed mutagenesis was performed to generate five mutated plasmids: pEGFP-C1-Q221P, pEGFP-C1-R224C, pEGFP-C1-S270 L, pEGFP-C1-G122*, and pEGFP-C1-A93Rfs*67. All plasmids were synthesized by the Beijing Genomic Institute (BGI, Beijing, China), and the entire sequence of the mutated constructs was confirmed by BGI.

### Cell culture, transfection, western blot, and qRT-PCR analysis

Human embryonic kidney 293T cells were cultured in Dulbecco’s modified Eagle’s medium (Invitrogen, Grand Island, NY, USA) supplemented with 10% foetal bovine serum and 2 mmol·L^*−*1^ L-glutamine in the presence of 5% CO_2_. Transient transfection was performed using Lipofectamine 3000 (Invitrogen) according to the manufacturer’s instructions. Forty-eight hours after transfection, the cells were collected and lysed with protein lysis buffer (Beyotime Technology, Shanghai, China) containing protease inhibitor cocktail (Solarbio, Beijing, China). Proteins were quantified using the bicinchoninic acid protein concentration assay kit (Invitrogen). Western blot analysis was performed with cell lysates containing 20 μg of total protein. After electrophoresis on a 10% polyacrylamide gel, the proteins were electrophoretically transferred to a polyvinylidene fluoride membrane and incubated with anti-GFP and anti-β-actin mouse polyclonal antibodies (Abcam, Cambridge, UK). The membrane was washed and incubated with peroxidase-conjugated goat anti-mouse secondary antibodies (Abcam).

In addition, total RNA was extracted from the transfected 293T cells using the TRIzol (Carlsbad, CA, USA) method, and 1 μg of the extracted RNA was used to synthesize cDNA with a reverse transcription kit (TaKaRa, Tokyo, Japan) according to the manufacturer’s protocol. qRT-PCR was performed on an ABI 7500 system (Applied Biosystems, CA, USA) using 2× Universal SYBR Green Fast qPCR Mix (ABclonal, Wuhan, China). The reaction conditions were 95 °C for 30 s during the initial hold, followed by 40 amplification cycles at 95 °C for 10 s and annealing for 15 s at 60 °C. The relative gene expression was normalized against the gene expression of *GAPDH* and calculated by the 2^−ΔΔCt^ method. The primers for the human *MSX1* gene were F: 5’-GACATGACTTCTTTGCCACTCG-3’ and R: 5’-CAGGAGCGAAGGGGACACTTT-3’. All experiments were performed in triplicate.

### Subcellular localization assay

After seeding on slides, 293T cells were transiently transfected with pEGFP-C1 expression plasmids containing GFP-wild-type or mutated MSX1 cDNA fusion proteins. At 48 h post-transfection, the cells were washed three times with phosphate-buffered saline and fixed with 4% paraformaldehyde for 15 min. After three washes with phosphate-buffered saline, the cells were placed in mounting medium with 4’,6-diamidino-2-phenylindole (Solarbio, Beijing, China), mounted, and photographed using an LSM 510 Meta confocal microscope (Zeiss, Oberkochen, Germany) with a ×40/1.00 numerical aperture oil objective lens.

### Statistical analysis

For verification of the nonsyndromic TA pattern in patients with *MSX1* variants, eight patients with detailed missing tooth site records from this study and previously published studies were included. According to the HGMD and PubMed databases, 36 MSX1 variants from 31 studies were identified, and 93 patients with detailed documentation of TA sites were assessed (Supplementary Table 1). Therefore, 101 patients were included in the study. Statistical analysis was performed using the GraphPad Prism software (v.8.0). A one-way chi-square test (or Fisher’s exact test) was used to compare the differences between tooth positions. Data are presented as the mean ± standard deviation (SD) (*n* = 3), and *P* < 0.05 was considered statistically significant. The *P* value style was acquired from GraphPad: NS, *P* = 0.123 4, *P = 0.033 2, **P = 0.002 1, ***P = 0.000 2, and ****P < 0.000 1.

## Supplementary information

Supplementary table 1

Supplementary figure 1

Supplementary figure 2

Supplementary figure 3

Supplementary figure 4
