# Supplementary material for: Novel MSX1 variants identified in families with nonsyndromic oligodontia
Source: Int J Oral Sci. 2021 Jan 8;13:2. doi: 10.1038/s41368-020-00106-0 (PMC7794556; doi:10.1038/s41368-020-00106-0)
Supplement: Supplementary file 1 — Supplementary table 1 [file 41368_2020_106_MOESM1_ESM.docx]

Supplement table 1. Summary of human MSX1 variations reported in HGMD and PubMed database

| Exon | Nucleocide change | Protein change | Type of variation | Reference |  | Right quadrants | | | | | | | | Left quadrants | | | | | | | |
| --- | --- | --- | --- | --- | --- | --- | --- | --- | --- | --- | --- | --- | --- | --- | --- | --- | --- | --- | --- | --- | --- |
|  |  |  |  |  | Max | 8 | 7 | 6 | 5 | 4 | 3 | 2 | 1 | 1 | 2 | 3 | 4 | 5 | 6 | 7 | 8 |
|  |  |  |  |  | Mand | 8 | 7 | 6 | 5 | 4 | 3 | 2 | 1 | 1 | 2 | 3 | 4 | 5 | 6 | 7 | 8 |
| 1 | c.95C>T | p.A32V | missense mutation | Ceyhan (2014) Indian J Dent 5, 172 |  | / | * | * | * | * |  | * |  |  | * |  | * | * | * | * | / |
|  |  |  |  |  |  | / |  | * | * |  |  |  |  |  |  |  |  | * | * | * | / |
| 1 | c.200T>A | p.M67K | missense mutation | Lidral (2002) J Dent Res 81, 274 |  | Wearing maxillary denture (excluding statistic analysis) | | | | | | | | | | | | | | | |
|  |  |  |  |  |  | * |  |  | * |  |  | * |  |  | * |  |  | * |  | * | * |
|  |  |  |  |  |  | * |  |  | * | * |  |  |  |  |  |  | * | * |  |  | * |
|  |  |  |  |  |  | * |  |  |  |  |  | * |  |  | * |  |  |  |  |  | * |
|  |  |  |  |  |  | * |  |  | * | * |  |  |  |  |  |  | * | * |  |  | * |
|  |  |  |  |  |  |  |  |  | * |  |  |  |  |  |  |  |  | * |  |  |  |
|  |  |  |  |  |  | * |  |  | * | * |  | * |  |  | * |  | * | * |  |  | * |
|  |  |  |  |  |  | * | * |  | * | * |  | * |  |  | * |  | * | * |  |  | * |
|  |  |  |  |  |  | * |  |  | * | * |  |  |  |  |  |  | * | * |  |  | * |
|  |  |  |  |  |  | * |  |  | * | * |  |  |  |  |  |  | * | * |  |  | * |
| 1 | c.348C>T | p.G116= | synonymous mutation | Qin (2013) Arch Oral Biol 58, 1180 |  | * |  |  | * |  | * | * |  |  | * |  | * | * |  |  | * |
|  |  |  |  |  |  | * |  |  | * |  |  | * | * | * | * |  | * | * |  |  | * |
|  |  |  |  |  |  | * |  |  | * | * |  | * |  |  | * |  | * | * |  |  | * |
|  |  |  |  |  |  | * |  |  | * | * |  |  | * | * |  |  | * | * |  |  | * |
| 1 | c.434G>A | p.W145* | nonsense mutation | Kimura (2014) Eur J Oral Sci 122, 15 |  | * | * |  | * | * |  |  |  |  |  |  | * | * |  | * | * |
|  |  |  |  |  |  | * | * | * | * |  |  |  | * | * |  |  |  | * | * | * | * |
|  |  |  |  |  |  |  |  |  | * | * |  |  |  |  |  |  | * | * |  |  | * |
|  |  |  |  |  |  | * |  |  | * |  |  |  | * | * |  |  |  | * |  |  | * |
|  |  |  |  |  |  |  |  |  | * | * |  |  |  |  |  |  |  | * |  |  |  |
|  |  |  |  |  |  | * |  |  | * |  |  |  |  |  |  |  |  | * |  |  | * |

| 1 | c.464C>A | p.P155Q | missense mutation | Ceyhan (2014) Indian J Dent 5, 172 |  | / |  |  | * |  |  | * |  |  | * |  |  | * |  |  | / |
| --- | --- | --- | --- | --- | --- | --- | --- | --- | --- | --- | --- | --- | --- | --- | --- | --- | --- | --- | --- | --- | --- |
|  |  |  |  |  |  | / |  |  | * |  |  |  |  |  |  |  |  | * |  |  | / |
|  |  |  |  |  |  | / |  |  | * |  |  |  |  |  |  |  |  | * |  |  | / |
|  |  |  |  |  |  | / |  |  |  |  |  |  |  |  |  |  |  | * |  |  | / |
| 2 | c.476T>G | p.L159R | missense mutation | Mu (2013) Genet Mol Res 12, 4446 |  | * |  |  | * | * |  | * |  |  | * |  | * | * |  |  | * |
|  |  |  |  |  |  | * | * |  | * |  |  | * |  |  | * |  |  | * |  | * | * |
|  |  |  |  |  |  | ? | * |  | * | * |  | * |  |  | * |  | * | * | * |  | ? |
|  |  |  |  |  |  | ? | * | * | * |  |  |  | * | * |  |  |  | * | * | * | ? |
|  |  |  |  |  |  | * | * |  | * | * |  | * |  |  |  |  |  | * |  | * | * |
|  |  |  |  |  |  | * | * |  | * |  |  |  | * | * |  |  | * | * |  | * | * |
|  |  |  |  |  |  | ? |  |  | * |  |  | * |  |  | * |  |  | * |  |  | ? |
|  |  |  |  |  |  | ? |  | * | * |  |  |  | * | * |  |  |  |  | * |  | ? |
|  |  |  |  |  |  | ? |  |  | * |  |  | * |  |  | * |  |  | * |  |  | ? |
|  |  |  |  |  |  | ? |  |  |  |  |  | * | * | * |  |  |  |  |  |  | ? |
|  |  |  |  |  |  | * |  |  |  |  |  |  | * | * |  |  |  |  |  |  | * |
|  |  |  |  |  |  | * |  |  |  |  |  |  |  |  |  |  |  |  |  |  | * |
| 2 (HD) | c.517C>A | p.R173S | missense mutation | Zhang (2015) Chin J Dent Res 18, 229 |  | * | * |  | * |  |  | * | * |  |  |  |  |  |  | * | * |
|  |  |  |  |  |  | * |  |  |  |  |  |  | * | * |  |  |  |  |  | * | * |
|  |  |  |  |  |  | * |  |  |  |  |  |  |  |  |  |  |  |  |  |  | * |
|  |  |  |  |  |  | * |  |  |  |  |  |  |  |  |  |  |  |  |  |  | * |
| 2 (HD) | c.526C>T | p.R176W | missense mutation | Bergendal (2011) Am J Med Genet A |  | / |  |  | * | * |  |  |  |  | * |  |  | * |  |  | / |
|  |  |  |  |  |  | / |  |  | * |  |  |  |  |  | * |  |  | * |  |  | / |
| 2 (HD) | c.539C>T | p.T180I | missense mutation | Yamaguchi (2014) PLoS One 9, e102944 |  | * | * |  | * |  |  |  |  |  |  |  | * | * |  | * | * |
|  |  |  |  |  |  | * |  |  |  |  |  |  |  |  |  |  |  |  |  |  | * |
| 2 (HD) | c.572T>C | p.F191S | missense mutation | Yang L (2020) PloS One 15(1): e0227287 |  | * | * |  | * | * |  |  |  |  |  |  | * | * |  | * | * |
|  |  |  |  |  |  | * | * |  |  | * |  |  |  |  |  |  | * |  |  | * | * |

| 2 (HD) | c.577C>T | p.Q193* | nonsense mutation | De Muynck (2004) Am J Med Genet 128A, 401 |  | * |  |  | * | * |  |  |  |  |  |  | * | * |  |  | * |
| --- | --- | --- | --- | --- | --- | --- | --- | --- | --- | --- | --- | --- | --- | --- | --- | --- | --- | --- | --- | --- | --- |
|  |  |  |  |  |  | * | * | * | * |  |  | * |  |  | * |  |  | * | * | * | * |
|  |  |  |  |  |  | * | * |  | * | * |  | * |  |  | * |  | * | * |  | * | * |
|  |  |  |  |  |  | * | * |  | * |  |  | * |  |  | * |  |  | * | * | * | * |
| 2 (HD) | c.583C>T | p.Q195* | nonsense mutation | Liang (2012) Eur J Oral Sci 120, 278 |  | * | * |  | * | * |  | * |  |  | * |  | * | * |  | * | * |
|  |  |  |  |  |  | * | * |  | * |  |  |  | * | * |  |  |  | * |  | * | * |
|  |  |  |  |  |  | * | * |  | * | * |  | * |  |  | * |  | * | * |  | * | * |
|  |  |  |  |  |  | * | * |  | * |  |  |  | * | * |  |  |  | * |  | * | * |
| 2 (HD) | c.599C>T | p.A200V | missense mutation | Mostowska (2006) J Appl Genet 47, 159 |  | * |  |  | * | * |  |  |  |  |  |  | * |  |  |  | * |
|  |  |  |  |  |  | * | * |  | * |  |  |  | * | * |  |  | * | * |  | * | * |
| 2 (HD) | c.605G>C | p.R202P | missense mutation | Vastardis (1996) Nat Genet 13, 417 |  | * |  |  | * | * |  | * |  |  |  |  | * | * |  |  | * |
|  |  |  |  |  |  | * |  |  | * |  |  |  |  |  |  |  |  | * |  |  | * |
|  |  |  |  |  |  | * |  |  | * | * |  |  |  |  |  |  | * | * |  |  | * |
|  |  |  |  |  |  | * |  |  | * |  |  |  |  |  |  |  |  | * |  |  | * |
|  |  |  |  |  |  | * |  |  | * |  |  |  |  |  |  |  |  | * |  |  | * |
|  |  |  |  |  |  | * |  |  | * |  |  |  |  |  |  |  |  | * |  |  | * |
|  |  |  |  |  |  | * |  |  | * |  |  |  |  |  |  |  |  | * |  |  | * |
|  |  |  |  |  |  | * |  | * | * |  |  |  |  |  |  |  |  | * | * |  | * |
|  |  |  |  |  |  | * |  |  | * | * |  |  |  |  |  |  | * | * |  |  | * |
|  |  |  |  |  |  | * |  |  | * |  |  |  |  |  |  |  |  | * |  |  | * |
|  |  |  |  |  |  | * |  |  | * | * |  |  |  |  |  |  | * | * |  |  | * |
|  |  |  |  |  |  | * |  | * | * |  |  |  |  | * |  |  |  | * | * |  | * |
|  |  |  |  |  |  | * |  |  | * | * |  | * |  |  | * |  | * | * |  |  | * |
|  |  |  |  |  |  | * |  | * | * |  |  |  |  | * |  |  |  | * | * |  | * |
|  |  |  |  |  |  | * |  |  | * | * |  |  |  |  |  |  |  | * |  |  | * |
|  |  |  |  |  |  | * |  | * | * |  |  |  |  |  |  |  |  | * | * |  | * |
|  |  |  |  |  |  | ? |  |  | * |  |  |  |  |  |  |  |  | * |  |  | ? |
|  |  |  |  |  |  | ? |  | * |  |  |  |  |  |  |  |  |  | * | * |  | ? |

| 2 (HD) | c.610G>T | p.E204* | nonsense mutation | Bonczek O (2018) Plos One |  | * |  |  | * | * |  | * |  |  | * |  | * | * |  |  | * |
| --- | --- | --- | --- | --- | --- | --- | --- | --- | --- | --- | --- | --- | --- | --- | --- | --- | --- | --- | --- | --- | --- |
|  |  |  |  |  |  | * |  |  | * |  |  |  | * |  |  |  |  | * |  |  | * |
|  |  |  |  |  |  | * | * |  | * | * |  | * |  |  | * |  | * | * |  | * | * |
|  |  |  |  |  |  | * | * |  | * |  |  |  |  | * |  |  |  | * |  | * | * |
| 2 (HD) | c.632T>G | p.L211R | missense mutation | Yamaguchi (2014) PLoS One 9, e102944 |  | ? | * | * |  | * |  |  | * | * | * |  | * |  | * |  | ? |
|  |  |  |  |  |  | ? |  | * | * | * | * |  | * | * |  | * | * | * | * |  | ? |
|  |  |  |  |  |  | * |  |  | * |  |  | * |  |  |  | * |  | * |  |  | * |
|  |  |  |  |  |  | * |  |  | * | * |  |  |  |  |  |  | * | * |  |  | * |
|  |  |  |  |  |  | * |  |  | * | * |  |  |  |  |  |  | * | * |  |  | * |
|  |  |  |  |  |  | * |  |  | * | * |  | * |  |  |  |  | * | * |  |  | * |
| 2 (HD) | c.668G>T | p.R223L | missense mutation | van den Boogaard (2012) J Med Genet |  | cannot find more details | | | | | | | | | | | | | | | |
| 2 (HD) | c.C667G | p.R223G | missense mutation | Ma T (2020) Ann Hum Genet 84(1): 97-101 |  | * |  |  | * | * |  |  |  |  |  |  | * | * |  |  | * |
|  |  |  |  |  |  | * | * | * | * |  | * |  | * | * |  | * |  | * | * |  | * |
| 2 (HD) | c.673G>A | p.A225T | missense mutation | Chishti (2006) J Hum Genet 51, 872 |  | * | * | * | * | * |  |  | * |  | * |  | * | * | * | * | * |
|  |  |  |  |  |  | * | * | * | * | * |  |  | * |  |  |  | * | * | * | * | * |
|  |  |  |  |  |  | * |  |  |  |  |  |  | * | * |  |  |  |  |  |  |  |
|  |  |  |  |  |  |  | * | * | * |  |  | * | * | * | * |  |  | * | * | * | * |
| 2 (HD) | c.680C>A | p.A227E | missense mutation | Xuan (2008) Arch Oral Biol 53, 773 |  | * | * |  | * |  | * | * |  |  | * | * |  |  |  | * | * |
|  |  |  |  |  |  | * |  |  | * |  |  |  |  |  |  |  |  | * |  |  | * |
|  |  |  |  |  |  | * |  |  |  |  | * | * | * | * | * |  |  |  |  |  | * |
|  |  |  |  |  |  | * |  |  | * |  | * | * | * | * | * | * |  | * |  |  | * |

| 2 (HD) | c.689T>C | p.L230P | missense mutation | Mostowska (2012) Arch Oral Biol 57, 790 |  | * |  |  | * |  |  |  |  |  |  |  |  | * |  |  | * |
| --- | --- | --- | --- | --- | --- | --- | --- | --- | --- | --- | --- | --- | --- | --- | --- | --- | --- | --- | --- | --- | --- |
|  |  |  |  |  |  | * |  |  |  |  |  |  |  |  |  |  |  |  |  |  | * |
|  |  |  |  |  |  | ? |  |  | * |  |  |  |  |  |  |  |  | * |  |  | ? |
|  |  |  |  |  |  | ? |  |  | * |  |  |  |  |  |  |  |  | * |  |  | ? |
|  |  |  |  |  |  | * |  |  | * |  |  |  |  |  |  |  |  | * |  |  | * |
|  |  |  |  |  |  | * |  |  | * |  |  |  |  |  |  |  |  | * |  |  | * |
|  |  |  |  |  |  | * |  |  | * |  |  |  |  |  |  |  |  | * |  |  | * |
|  |  |  |  |  |  | * |  |  | * |  |  |  |  |  |  |  |  | * |  |  | * |
|  |  |  |  |  |  | * |  |  | * |  |  |  |  |  |  |  |  | * |  |  | * |
|  |  |  |  |  |  | * |  |  | * |  |  |  |  |  |  |  |  | * |  |  | * |
| intron | c.469+46_469+56delGCCGGGTGGGG |  | frameshift mutation | Pawlowska (2009) Tohoku J Exp Med 217(4): 307-12 |  |  |  |  | * |  |  |  |  |  |  |  |  | * |  |  | ? |
|  |  |  |  |  |  |  |  |  | * | * |  |  |  |  |  |  | * | * |  |  |  |
|  |  |  |  |  |  | * |  |  |  |  |  | * |  |  | * |  |  |  |  |  | * |
|  |  |  |  |  |  | * |  |  | * |  |  |  | * | * |  |  |  | * |  |  | * |
| 1 | c.80dupG | p.G27Rfs*168 | frameshift mutation | Kim (2006) J Dent Res 85, 267 |  | * |  |  | * |  |  |  |  |  |  |  |  | * |  |  | * |
|  |  |  |  |  |  | * |  |  | * |  |  |  | * | * |  |  |  | * |  |  | * |
|  |  |  |  |  |  | ? |  |  | * | * |  | * |  |  | * |  | * | * |  |  | ? |
|  |  |  |  |  |  | ? | * |  | * |  |  |  | * | * |  |  |  | * |  | * | ? |
| 1 | c.128_147del20 | p.M43Sfs*125 | frameshift mutation | Xin T (2018) Stem Cell Res Ther 9(1): 221 |  | * |  |  | * |  |  |  |  |  |  |  |  | * |  |  | * |
|  |  |  |  |  |  | * |  |  |  |  |  |  |  |  |  |  |  |  |  |  | * |
|  |  |  |  |  |  | * |  |  | * | * |  |  |  |  |  |  | * | * |  |  | * |
|  |  |  |  |  |  | * |  |  | * |  |  |  |  |  |  |  |  | * |  |  | * |
|  |  |  |  |  |  |  |  |  | * | * |  |  |  |  |  |  | * | * |  |  |  |
|  |  |  |  |  |  | * |  |  | * |  |  |  |  |  |  |  |  | * |  |  |  |
|  |  |  |  |  |  |  |  |  | * | * |  |  |  |  |  |  | * | * |  |  | * |
|  |  |  |  |  |  | * |  | * | * |  |  |  |  |  |  |  |  | * | * |  | * |
|  |  |  |  |  |  | * |  |  | * | * |  |  |  |  |  |  | * | * |  |  | * |
|  |  |  |  |  |  | * |  |  | * |  |  |  |  |  |  |  |  | * |  |  | * |
|  |  |  |  |  |  | * |  |  | * | * |  | * |  |  |  |  | * | * |  |  | * |
|  |  |  |  |  |  | * |  |  | * |  |  |  |  |  |  |  |  | * |  |  | * |
| 1 | c.249delC | p.E84Rfs | frameshift mutation | Prasad (2016) J Med Genet 53, 98 |  |  |  |  | * | * |  |  |  |  |  |  | * | * |  |  |  |
|  |  |  |  |  |  |  |  | * | * |  |  |  |  |  |  |  |  | * | * |  |  |
| 2 (HD) | c.566_572dupGCAAGTT | p.F191fs | frameshift mutation | Abid (2017) Arch Oral Biol 75, 8 |  | * |  | * | * | * |  |  |  |  |  |  | * | * | * |  | * |
|  |  |  |  |  |  | * |  | * | * | * |  |  |  |  |  |  |  | * | * |  | * |
|  |  |  |  |  |  | * |  | * | * |  |  |  |  |  |  |  |  | * | * |  | * |
|  |  |  |  |  |  | * |  | * | * |  |  |  |  |  |  |  | * | * | * |  | * |
| 2 (HD) | c.590_594dupTGTCC | p.L197Sfs*22 | frameshift mutation | Yang L (2020) PloS One 15(1): e0227287 |  |  | * |  | * | * | * | * | * | * | * | * | * |  |  | * | * |
|  |  |  |  |  |  | * | * |  | * |  |  | * | * | * | * | * |  | * |  | * | * |
| 2 (HD) | c.644dupA | p.Q215Qfs*125 | frameshift mutation | Bergendal (2011) Am J Med Genet A |  | / | * | * | * | * |  | * |  |  | * |  | * | * | * | * | / |
|  |  |  |  |  |  | / | * | * | * |  |  |  | * | * |  |  |  | * | * | * | / |
| 2 (HD) | c.665dupA | p.N222Kfs*118 | frameshift mutation | Arte (2013) PLoS One 8, e73705 |  | * |  |  | * | * |  |  |  |  | * |  | * | * |  |  |  |
|  |  |  |  |  |  | * | * |  | * |  |  |  | * | * |  |  |  | * |  | * | * |
| 2 | c.708delG | p.K237Sfs*2 | frameshift mutation | Arte (2013) PLoS One 8, e73705 |  | * |  |  | * | * |  |  |  |  |  |  | * | * |  |  | * |
|  |  |  |  |  |  | * |  |  | * |  |  |  | * | * |  |  |  | * |  |  | * |
| 2 | c.741_750dupACCGGCTGCC | p.F251Pfs*92 | frameshift mutation | AlFawaz (2015) Arch Oral Biol 60, 982 |  | * |  |  | * |  |  |  |  |  |  |  |  | * |  |  | * |
|  |  |  |  |  |  | * |  |  | * |  |  |  |  |  |  |  |  | * |  |  | * |
|  |  |  |  |  |  |  |  |  | * |  |  |  |  |  |  |  |  | * |  |  |  |
|  |  |  |  |  |  | * |  |  | * |  |  |  |  |  |  |  |  | * |  |  | * |
|  |  |  |  |  |  | * |  |  | * | * |  |  |  |  |  |  | * | * |  |  | * |
|  |  |  |  |  |  | * |  |  | * |  |  |  |  |  |  |  |  | * |  |  | * |
|  |  |  |  |  |  |  |  |  | * | * |  |  |  |  |  |  |  | * |  |  |  |
|  |  |  |  |  |  | * |  |  | * |  |  |  |  |  |  |  |  | * |  |  | * |
|  |  |  |  |  |  | * |  |  | * |  |  |  |  |  |  |  |  | * |  |  | * |
|  |  |  |  |  |  | * |  |  | * |  |  |  |  |  |  |  |  | * |  |  | * |
|  |  |  |  |  |  |  |  |  | * |  |  |  |  |  |  |  |  | * |  |  |  |
|  |  |  |  |  |  |  |  |  | * |  |  |  |  |  |  |  |  | * |  |  |  |
|  |  |  |  |  |  |  |  |  | * |  |  |  |  |  |  |  |  | * |  |  |  |
|  |  |  |  |  |  |  |  |  | * |  |  |  |  |  |  |  |  | * |  |  |  |

| 2 | c.844delG | p.Ala282Argfs*21 | frameshift mutation | Mitsui (2016) Sci Rep 6, 38398 |  |  |  |  | * | * |  |  |  |  |  |  | * | * |  |  |  |
| --- | --- | --- | --- | --- | --- | --- | --- | --- | --- | --- | --- | --- | --- | --- | --- | --- | --- | --- | --- | --- | --- |
|  |  |  |  |  |  | * |  |  |  |  |  |  |  | * |  |  |  | * |  |  | * |
|  |  |  |  |  |  |  |  |  | * | * |  |  |  |  |  |  | * | * |  |  | * |
|  |  |  |  |  |  | * |  |  | * |  |  | * |  |  | * |  |  | * |  |  | * |
| 2 | c.910_911dupTA | p.X304Yext*48 | nonstop mutation | Wong (2014) Mutagenesis 29, 319 |  | * |  |  | * | * |  | * | * |  | * | * | * | * |  |  | * |
|  |  |  |  |  |  | * | * |  | * |  |  | * | * | * |  | * |  | * |  | * | * |
|  | c.470-9G>A | IVS1 as G-A -9 | splicing | Tatematsu (2015) PLoS One 10,e0128227 |  |  |  |  | * |  |  |  |  |  |  |  |  | * |  |  |  |
|  |  |  |  |  |  |  |  |  |  | * |  |  |  |  |  |  |  | * |  |  |  |
|  |  |  |  |  |  | * | * |  | * | * |  | * | * |  |  | * |  | * |  | * | * |
|  |  |  |  |  |  |  | * | * |  |  | * |  |  |  |  |  | * |  | * | * | * |
|  |  |  |  |  |  | * |  |  | * |  |  |  |  |  |  |  |  | * |  |  |  |
|  |  |  |  |  |  | * |  |  | * |  |  |  | * | * |  |  |  | * | * | * | * |
|  |  |  |  |  |  | * |  |  | * |  |  |  |  |  |  |  |  | * |  |  | * |
|  |  |  |  |  |  | * | * |  | * |  |  | * |  | * |  |  |  | * |  |  | * |
|  | c.470-2A>G | IVS1 as A-G -2 | splicing | Li (2008) Zhonghua Kou Qiang Yi Xue Za Zhi 43, 157 |  | cannot find more details | | | | | | | | | | | | | | | |

|  | c.469+5G>A | IVS1 ds G-A +5 | splicing | Xue (2016) Clin Chim Acta 461, 135 |  | / | * | * |  |  | * |  |  |  |  |  | * |  | * | * | / |
| --- | --- | --- | --- | --- | --- | --- | --- | --- | --- | --- | --- | --- | --- | --- | --- | --- | --- | --- | --- | --- | --- |
|  |  |  |  |  |  | / | * | * | * |  |  |  |  |  |  | * | * | * |  | * | / |
|  |  |  |  |  |  | / |  |  | * | * | * | * |  |  |  | * | * | * |  |  | / |
|  |  |  |  |  |  | / |  |  | * | * | * | * | * | * | * | * |  | * |  |  | / |
|  |  |  |  |  |  | / | * | * |  |  |  |  |  |  |  |  |  | * | * | * | / |
|  |  |  |  |  |  | / | * | * |  |  |  |  | * |  |  |  |  | * | * | * | / |
|  |  |  |  |  |  | / | * |  |  | * |  |  |  | * |  |  |  |  |  | * | / |
|  |  |  |  |  |  | / | * |  |  |  |  |  |  |  |  |  |  |  | * | * | / |
|  |  |  |  |  |  | / |  |  |  | * |  |  |  |  |  |  | * |  |  | * | / |
|  |  |  |  |  |  | / | * |  |  | * |  |  |  |  |  |  | * |  | * | * | / |
|  |  |  |  |  |  | / | * |  |  | * |  |  |  |  |  |  | * |  |  | * | / |
|  |  |  |  |  |  | / | * |  |  |  |  |  | * |  |  |  | * |  |  | * | / |
|  |  |  |  |  |  | / |  |  |  |  |  |  |  |  |  |  |  |  |  |  | / |
|  |  |  |  |  |  | / |  |  |  |  |  | * | * |  |  |  |  |  |  |  | / |
|  |  |  |  |  |  | / |  |  | * |  |  |  |  |  |  |  |  | * |  |  | / |
|  |  |  |  |  |  | / |  | * | * |  |  |  |  |  |  |  |  | * |  |  | / |
|  |  |  |  |  |  | / |  |  | * | * | * |  | * | * |  |  | * | * |  |  | / |
|  |  |  |  |  |  | / | * | * | * | * |  |  |  | * | * |  | * | * |  |  | / |
|  |  |  |  |  |  | / | * |  |  |  |  |  |  | * |  |  |  |  |  | * | / |
|  |  |  |  |  |  | / | * |  |  |  |  |  |  |  |  |  |  |  | * |  | / |

Note: *: congenital missing teeth; Max: Maxillary; Mand: Mandibular; /: excluding teeth; ?: impossible diagnosis for young age patients;
 2(HD): Homeodomain; 2: outside the Homeodomain
