# Supplementary figures and images for: Novel MSX1 variants identified in families with nonsyndromic oligodontia
Source: Int J Oral Sci. 2021 Jan 8;13:2. doi: 10.1038/s41368-020-00106-0 (PMC7794556; doi:10.1038/s41368-020-00106-0)

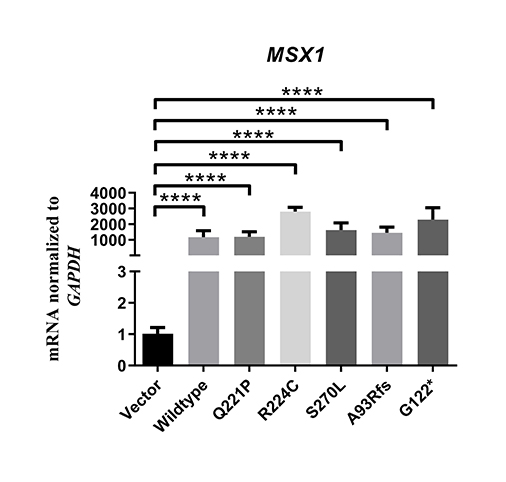

Supplement: Supplementary file 2 — Supplementary figure 1 [file 41368_2020_106_MOESM2_ESM.jpg]

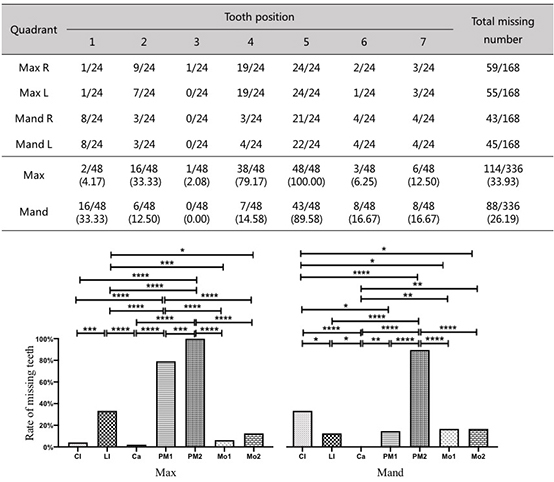

Supplement: Supplementary file 3 — Supplementary figure 2 [file 41368_2020_106_MOESM3_ESM.jpg]

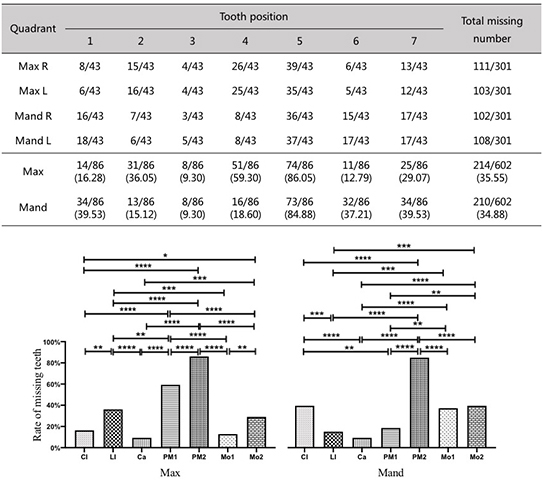

Supplement: Supplementary file 4 — Supplementary figure 3 [file 41368_2020_106_MOESM4_ESM.jpg]

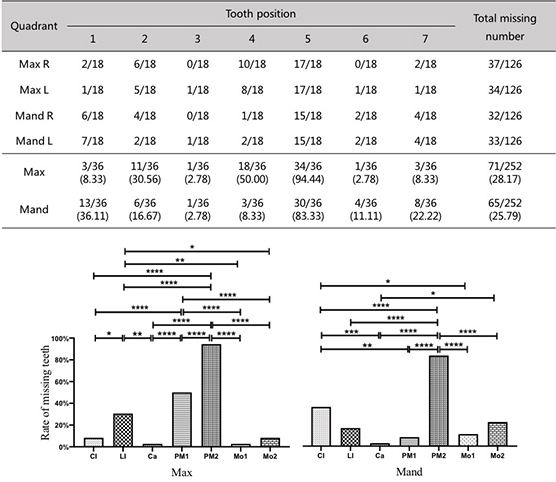

Supplement: Supplementary file 5 — Supplementary figure 4 [file 41368_2020_106_MOESM5_ESM.jpg]
